# Optimal Education: Paving the Way for True Optimal Start Dialysis

**DOI:** 10.1016/j.xkme.2025.101151

**Published:** 2025-10-16

**Authors:** Maria Camila Bermudez, Ankur D. Shah, Osama El Shamy

**Affiliations:** 1 Department of Nephrology, Geisinger Health, Danville, PA; 2 Warren Alpert Medical School of Brown University, Providence, RI; 3 Division of Kidney Disease and Hypertension, Department of Medicine, Rhode Island Hospital, Providence, RI; 4 Division of Kidney Disease and Hypertension, George Washington University, Washington, DC

**Keywords:** Dialysis, education, optimal

## Abstract

The comprehensive kidney care contracting program defines Optimal End Stage Renal Disease Starts as new patients with end-stage kidney disease who receive a preemptive kidney transplant, home dialysis, or initiating in-center hemodialysis using an arteriovenous access. These optimal starts are not possible without optimal patient education. Because policy changes make treatment options become available regardless of diagnosis (acute or chronic kidney injury) and setting, education must similarly evolve to be both site-independent and diagnosis-independent. In this piece, we explore gaps in the delivery of kidney failure modality education across the outpatient nephrology clinics, and dialysis units, and inpatient setting. We highlight both national and international models in each that have proven successful, the important role of both urgent-start peritoneal dialysis and transitional care units, and provide a roadmap for the successful implementation of an inpatient kidney failure educational program to help combat the reality of the high incidence of in-center hemodialysis among crash-start patients.

The persistent high rate of crash starts in patients initiating dialysis remains a critical challenge in kidney care delivery. Despite decades of quality improvement initiatives, ∼20% of patients starting dialysis do so without having ever seen a nephrologist, nearly 85% of patients start hemodialysis (HD) with a catheter, and in spite of the recognized benefits of home dialysis, only 14.1% of patients start with a home modality.^1^ These crash starts are associated with higher mortality, increased hospitalizations, and substantially higher costs than planned dialysis starts.^2^, ^3^, ^4^

In 2019, the comprehensive kidney care contracting program—part of the kidney care choices (KCCs) Model—set out to encourage and define optimal end-stage kidney disease starts. It is the percentage of new patients with end-stage kidney disease who received a preemptive kidney transplant, home dialysis, or initiating in-center HD using an arteriovenous access.^5^ These optimal starts are not possible without optimal patient education. Of note, the KCC model does provide benefit enhancements for kidney disease education, covering up to 6 1-hour sessions for patients with stage 4 chronic kidney disease (CKD), CKD stage 5 and the first 6 months of kidney failure.

Education is one of the strongest tools to overcome the strong bias toward in-center HD in the United States.^6^ This bias is particularly pronounced in urgent-start scenarios, in which many patients are defaulted to in-center HD, having never been educated on or offered home HD or peritoneal dialysis (PD). Current gaps in after kidney failure nephrology care mean that traditional office-based education alone is insufficient, but it is the only reimbursed option. However, the landscape of dialysis initiation is evolving, with a 2025 policy change allowing home dialysis for acute kidney injury (AKI) patients.^7^ This expansion of home therapy creates new opportunities while potentially exposing existing gaps in patient education across the continuum of patient care. Because treatment options become available regardless of diagnosis (AKI or CKD) and setting (clinic, hospital, or dialysis facility), education must similarly evolve to be both site-independent and diagnosis-independent.

## Conceptual Framework

**Figure 1:**
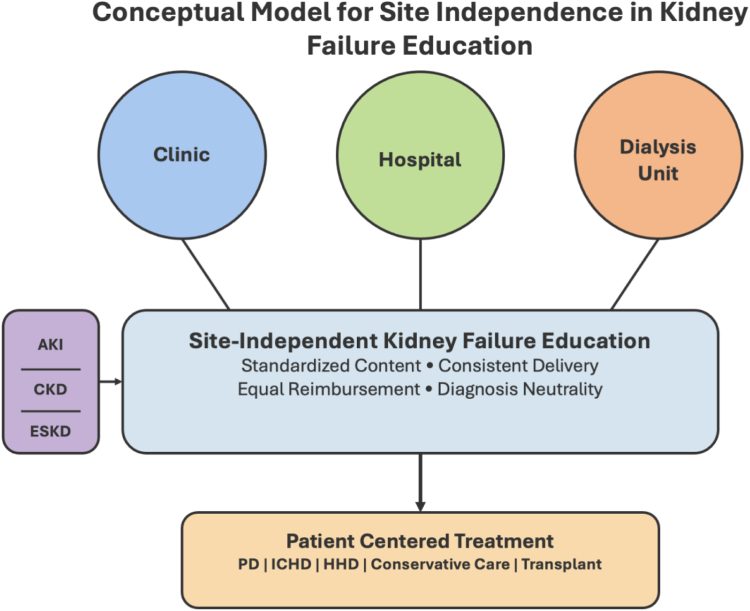
Conceptual model for site-independent dialysis education.

The core principle of site independence acknowledges that patient education should not be limited by location. Whether a patient encounters the health care system through a nephrology office, hospital admission, or dialysis facility, they deserve equal access to complete information about their treatment options. This comprehensive approach respects patient autonomy while acknowledging the practical realities of diverse care pathways (Fig 1).

Currently, reimbursement structures and care pathways are incomplete, only offering office-based education during a limited period of CKD progression. Patients with AKI who will, under the new policy change, be able to access home modalities are less likely to receive the same comprehensive education historically reserved for patients with CKD. This creates significant gaps for patients who enter the system through other channels, particularly those experiencing urgent or unplanned dialysis starts.

Although treatment options are expanding, our educational models have not kept pace. Patients who are diagnosed in the hospital have had limited access to home modalities. When considering educational programs and approaches, it is important to consider the setting and formulation of such initiatives. It has been shown that interactive, frequent, multifaceted educational interventions with both individual and group participation have the greatest potential at improving patient outcomes, knowledge, and management.^8^ Value-based companies such as Strive Health, Monogram Health, Evergreen Nephrology, and Panoramic Health have leveraged data analysis, risk stratification, and patient selection models to implement early interventions in patients with CKD. However, more needs to be done, especially with regard to bridging the aforementioned gaps in patient care.

## Outpatient Modality Education: Timing and Outcomes

Traditionally, a provider’s decision to initiate the modality education discussion was simply based on a patient’s estimated glomerular filtration rate value (<30mL/min/1.73m^2^). However, the most recent KDIGO guidelines^9^ recommend the use of externally validated models, such as the Kidney Failure Risk Equation and Kaiser Permanente Northwest model to determine the 2-year risk of kidney failure. Aside from the estimated glomerular filtration rate, these models take into consideration factors such as age, sex, diabetes status, blood pressure, albumin/creatinine ratio, hemoglobin, calcium, phosphate, and bicarbonate. If the risk is >40%, then modality education, access planning and transplant referral are all potential routes of action.

Styles and approaches to modality education differ based on both the provider and patient. There are 4 main types of approaches to patient education and shared decision-making commonly seen: institutionalist, paternalist, informative, and interpretive.^10^ The institutionalist provides education within the framework of institutional resources and policies, often influenced by practice culture, incentives, and patient preferences. The main focus here is on survival, quality of life, and patient education on dialysis modalities. The paternalist’s approach is mainly focused on their own perceived patient preferences and wellbeing, irrespective of the patient’s current preferences. The informative approach, although admirable, does not offer a treatment recommendation to patients unless explicitly requested to do so. Finally, the interpretive approach is the most complete. It guides the patients to their optimal option based on their goals, values, and wishes, while considering the broader network of helpers, support, and care partners. These variations in approaches dictate the patient experience and play a pivotal role in the decision-making process.

Modality education programs can be constructed in several ways: individual versus group education, physician-led versus advanced practice providers, nurse educators, and some offer peer support groups. Predialysis education has shown that patients’ preference for home dialysis far outpaces the current prevalence of home modalities, with as many as 74% of patients opting for a home modality.^11^, ^12^, ^13^ Furthermore, early education has been shown to reduce the incidence of dialysis initiation—presenting patients with alternatives such as preemptive kidney transplantation and conservative medical management.^14^ Multiple programs have reported successful implementation of educational programs, including Aloudah et al^15^ in Saudi Arabia, who instituted a monthly 2-hour class for all advanced patients with CKD. Follow-up calls over a year after the attending the class showed that 87% of the participants had started dialysis, 72% of whom were on HD and the rest were on PD. This is despite 41.9% preferring PD over HD in the event that they are not able to receive a preemptive kidney transplant. This discrepancy between modality preference and eventual outcome was also demonstrated in a single-center retrospective study in which cited reasons for this included lack of a care partner, lack of home space, and poor surgical candidacy.^13^

## Dialysis-Based Education

For patients who were not provided modality education in either the outpatient or inpatient settings that end up treated with dialysis, transitional care units (TCUs) provide an invaluable opportunity for hands-on learning and education.

Potential candidates for the TCU include new-start patients receiving in-center HD, kidney transplant recipients expected to initiate HD in the near future, and patient receiving PD who are at risk for modality change. Those are in hospice care, residents of long-term care facilities without a care partner, unstable living environment and significant cognitive impairments are poor candidates for the TCU.^16^ Unlike standard of care in the in-center HD units, the TCU offers patients the opportunity for more one-to-one care with staff. Typical programs are anywhere from 3-8 weeks long with an initial emphasis on identifying and addressing any physical and psychosocial issues a patient may be experiencing. Most TCU prescriptions are 4-5 days a week with blood and dialysate flow rates similar to those used in a typical home HD prescription. Ideally, the program’s home dialysis machine of choice is used in the TCU – thereby reducing training time if the patient opts for home HD because of familiarity with the machine.^17^

During a patient’s treatment time, the TCU staff have the opportunity to involve patients in their own treatment delivery, tailoring education to patients’ needs and learning style, as well as early engagement with the home dialysis staff.^18^

Integration of a TCU into the floor plan of a dialysis can be a: (1) separated, stand-alone unit; (2) integrated with the home therapies training area; (3) integrated throughout the in-center HD unit; and (4) combined home therapies training area and in-center HD.^15^ In a retrospective matched study,^19^ patients initiating dialysis in TCUs were 16% less likely to be on in-center HD at the 14-month mark and 2.5 times more likely to be on home dialysis. Other TCU programs^20^, ^21^, ^22^, ^23^, ^24^ demonstrated improved mortality, access placement, and home dialysis initiation, and anemia control and mineral bone disorder management. It is important to note that although the specifics of program implementation differed in duration and personnel, the general outline and educational program targets were very similar.

## Inpatient Modality Education: Unmet Needs and Integration

Although there is an emphasis on increasing modality education in the outpatient setting, an often overlooked gap is inpatient education. Given that up to 63% of patients crash start into dialysis,^25^^,^^26^ it comes as quite a surprise that we are not intercepting those patients more often in the inpatient setting. Although urgent-start dialysis is traditionally thought of as the placement of a temporary or tunneled central venous catheter, followed by initiation of HD sessions – sometimes with incrementally increasing blood and dialysate flow rates. A prior study found that 87% of patient starting acute dialysis remained on in-center HD at the time of discharge.^23^ However, there have been numerous studies demonstrating the feasibility and safety of implementing urgent-start PD programs.^27^, ^28^, ^29^ The implementation of an inpatient educational program in the aforementioned study resulted in a drop in the short-term in-center HD initiation rate to 65%.^23^

Using flipcharts and manuals, specially trained dialysis nurses provided a structured in-hospital education program across 3 hospitals in Germany.^30^ This resulted in a 15% increase in PD rates in unplanned dialysis starts and an overall increase of 66% across all dialysis starts. This demonstrates the great potential for an impact through focusing efforts on growing inpatient education. This requires a concerted effort, and an example of its successful implementation was demonstrated at Kaiser Permanente Northern California.^31^ Patient and family education was only one component of the integration plan. Health care professional and organizational education, operational improvements (including the development of urgent-start PD), as well monitoring and continuous quality improvement were all integral in the success of this program. PD initiation increased from 15.2% in 2008 to 33.8% in 2018, with 80.3% of those who were initiated on PD in 2018 remaining on the modality at the one-year mark.

## Blueprint for the Implementation of an Inpatient Kidney Failure Educational Program: Closing the Gap

Despite the aforementioned success in implementing inpatient modality education, a significant gap exists in the provision of dialysis education, with institutional variations in access and quality, yielding inconsistent and suboptimal treatment initiation. This paradox underscores the complex interplay of systemic, provider, and patient barriers that hinder the implementation of effective educational programs and optimal treatment starts.

Integrating dialysis options education into a hospital-based program fosters a collaborative relationship between patients, care partners, and the kidney failure education and care teams, promoting informed decision-making and seamless care transitions. We propose 6 pillars for a hospital-based kidney failure educational program (Table 1). Additionally, the pathway from modality preference to execution is complex, requiring thorough barrier assessment, timely access planning, and patient readiness evaluation. Delays in executing any of the necessary steps because of provider and patient inertia or rapid kidney function decline can lead to suboptimal starts. A dedicated hospital-based kidney failure program, with a multidisciplinary team dedicated to providing step-by-step guidance from education to coordination, can facilitate a seamless and efficient transition from preference to reality, ensuring optimal starts.

**Table 1:** The 6 Pillars of a Hospital-Based Kidney Failure Educational Program

Six Pillars
1. Universal Access: Ensuring equal access to education for all patients.
2. Informative: Providing unbiased information, without favoring one option over the others.
3. Promptness: Delivering education in a timely manner.
4. Tailoring: Customizing education to individual patient needs and preferences.
5. Flexibility: Adapting education to accommodate different learning styles.
6. Continuity: Ensuring seamless transitions and ongoing support.

*Note:* Conceptual model for site-independent dialysis education.

## Key Components of a Hospital-Based Educational Program

1. Systematic screening and enrollment: use electronic medical records to identify patients with advanced kidney disease, ensuring equitable access to educational programs and eliminating variability in provider referral thresholds and biases.
2. Risk-based pathways: develop tailored pathways based on the estimated need for imminent kidney replacement therapies, enabling expedited education and support for high-risk patients, personalized guidance, and seamless transitions to dialysis starts.
3. Multidisciplinary team: assemble a team of advanced kidney care experts, including a nurse educator and a home champion physician, to deploy educational materials, guide shared decision-making, and coordinate care.
4. Early screening of barriers to home dialysis: identify and address modifiable barriers, developing a plan to overcome them and reassess over time.
5. Standardized educational materials: provide high-quality, standardized materials catering to patient preferences, including telemedicine, individual/group training, and various learning styles and levels of literacy.
6. Electronic dashboard: implement an electronic dashboard to alert the team to new laboratories, kidney function decline, or hospitalizations, ensuring timely interventions and the need for re-education and realignment of therapy goals.
7. Coordination and monitoring: coordinate care, monitoring GFR decline and AKI episodes to expedite education, including 1:1 bedside sessions and facilitate optimal dialysis starts.
8. Goal-oriented care planning: develop patient-centered care plans prioritizing shared decision-making and goal-oriented outcomes.

By investing in internal programs, hospitals can harness the transformative power of education, cultivating a community of empowered patients, informed care partners, and care teams ultimately facilitating optimal starts and improved outcomes.

## Conclusion

The implementation of kidney failure educational programs across the continuum of patient care: inpatient, outpatient, and the dialysis unit, is an essential component of patient care. It is key in providing patients with a true optimal start into their journey. Efforts should be made to emphasize education and improve its reimbursement rates, thereby incentivizing health care systems to provide patients with the information they need to navigate their care.

It has been shown that educational programs may increase home dialysis uptake, resulting in reductions of health care costs to payors when compared with in-center HD, which is intriguing to both payors and the Centers for Medicare and Medicaid and resulted in the implementation of incentives to grow home dialysis care. However, this has the potential of influencing providers to enroll their patients in home modalities, even if they may not be ideal candidates. This could result in transitions to alternative modalities, something that has been shown to be significantly more costly to the health care system.

Incentivizing health care systems to provide optimal education to patients while honoring and supporting their ultimate treatment of choice irrespective of the short-term treatment costs is the true optimal path. It reduces the likelihood of transitions, which has mental, emotional, physical, and monetary effects on patients and the health care system.
